# Efficiency Assessment of Fenton-Based Pre-Treatment of Medical Wastewater Using Fe, Cu, and Mn Catalysts—Impact on the Aquatic Environment

**DOI:** 10.3390/molecules31061060

**Published:** 2026-03-23

**Authors:** Andrzej R. Reindl, Maciej Tankiewicz, Agnieszka Fiszka Borzyszkowska, Lidia Wolska

**Affiliations:** Department of Environmental Toxicology, Faculty of Health Sciences, Medical University of Gdansk, 80-210 Gdansk, Poland; maciej.tankiewicz@gumed.edu.pl (M.T.); agnieszka.fiszka_borzyszkowska@gumed.edu.pl (A.F.B.); lidiawolska@gumed.edu.pl (L.W.)

**Keywords:** Fenton oxidation, medical wastewater, pharmaceutical removal, ecotoxicity bioassays, environmental risk assessment

## Abstract

This study evaluated the efficiency and ecotoxicological impact of the Fenton oxidation process with different metal-based catalysts (FeSO_4_, CuSO_4_, MnSO_4_) in removing pharmaceuticals and organic contaminants from real hospital wastewater. All catalytic systems achieved high oxidation, with COD reduction reaching 81–89% after 4 h. Two complementary approaches were applied: targeted LC-MS/MS quantification of a model mixture of antibiotics and pharmaceuticals, and untargeted GC-MS/MS screening method for assessing the overall organic contaminant profile. Toxicity was assessed using Microtox^®^. Targeted analysis showed complete or near-complete degradation of β-lactams, tetracyclines and most sulfonamides, with slightly lower removal for sulfamethoxazole in FeSO_4_ system (96%). Fluoroquinolones and selected pharmaceuticals, such as caffeine and propranolol were more resistant, particularly with CuSO_4_ and MnSO_4_ catalysts. The untargeted GC-MS/MS screening revealed the highest overall reduction in chromatographic peak areas for FeSO_4_ (70%), followed by MnSO_4_ (39%) and CuSO_4_ (36%). GC-MS/MS profiling confirmed that the Fe-catalyzed process was the most effective in reducing the total chromatographic peak area (70%). However, ecotoxicological assays revealed a significant increase in toxicity post-treatment, with growth inhibition of *Allivibrio fischeri* reaching 98%. This suggests that high oxidation does not directly correlate with biological safety, likely due to the presence of unconsumed reagents or the formation of transformation products with higher acute toxicity. These findings emphasize the necessity of integrating bioassays into treatment evaluation protocols to assess the true environmental risk of treated effluents.

## 1. Introduction

The interaction of hospital wastewater with aquatic ecosystems poses significant ecological risks due to the presence of pharmaceuticals and the acceleration of antimicrobial resistance [1,2,3,4]. Previous studies have confirmed the detection of these compounds in various water bodies, raising public health concerns [5,6,7].

Traditional wastewater treatment methods frequently fail to adequately remove chemically complex pharmaceutical compounds. Consequently, advanced oxidation processes (AOPs) such as the Fenton process using Fe^2+^, Cu^2+^, or Mn^2+^ ions have garnered increasing interest for their high efficiency in degrading pharmaceuticals and other persistent organic pollutants by generating reactive hydroxyl radicals (•OH) [8,9,10].

However, chemical treatment can generate by-products with higher ecotoxicity than untreated effluents, mirroring the complexity of healthcare wastewater. The response of indicator organisms to these contaminants varies based on biological sensitivities, often evaluated using the Microtox^®^ assay [11,12]. Previously studies suggest that using organisms collected directly from natural environments, particularly from the aquatic ecosystem, for ecotoxicological tests may provide a more accurate reflection of real-world toxicity impacts. This approach could offer novel insights into the risks posed by chemical by-products, particularly those formed during disinfection processes such as ozonation and chlorination [13]. For instance, the intensified Fenton oxidation process has been investigated for hospital wastewater treatment, where short-chain organic acids, such as oxalic and formic acid, were identified as primary by-products, ultimately yielding non-toxic effluents [14]. However, alternative disinfection methods like peracetic acid and chlorine dioxide have been found to generate by-products that could pose risks to aquatic ecosystems. Thus, while chemical treatment methods can be effective in wastewater decontamination, their unintended ecotoxicological consequences must be carefully assessed to ensure that treatment processes do not exacerbate environmental hazards. Both treated and untreated wastewater contribute to increased nutrient levels in aquatic ecosystems, thereby promoting eutrophication. This process leads to excessive growth of macrophytes and phytoplankton, resulting in significant ecological consequences due to the presence of various chemical compounds in wastewater. Aquatic microorganisms may be particularly sensitive to toxic intermediates generated during Fenton treatment [15].

The aim of this study was to evaluate the efficiency of the Fenton oxidation process using different transition metal catalysts (FeSO_4_, CuSO_4_, MnSO_4_) in the degradation and removal of selected pharmaceutical residues from real wastewater streams. Particular emphasis was placed on the oxidation of various classes of antibiotics and pharmaceuticals, as well as on the resulting changes in ecotoxicity assessed using *Aliivibrio fischeri* bioluminescent bacteria (Microtox test). Additionally, attention was paid to the identification of oxidation products formed in real hospital wastewater samples, which may affect the ecotoxicological assessment.

The novelty of this work lies in the combined assessment of chemical degradation efficiency and ecotoxicological effects of post-treatment wastewater under real environmental conditions. Unlike many previous studies that rely on model solutions or synthetic wastewater, this research was based on actual wastewater samples from hospitals (with and without oncology departments) and municipal sewage systems collected in the Tri-City agglomeration area in Poland. Furthermore, this study compares multiple catalysts in the same experimental setup, highlighting differences not only in pollutant removal but also in their ecological safety post-treatment.

Although the experiments were performed at laboratory scale, they reflect practical scenarios encountered in decentralized treatment units or pre-treatment systems in medical facilities. It should be noted, however, that the findings are based on selected wastewater streams and do not account for potential seasonal or regional variability.

## 2. Results

### 2.1. Fenton Oxidation Results

**Figure 1:**
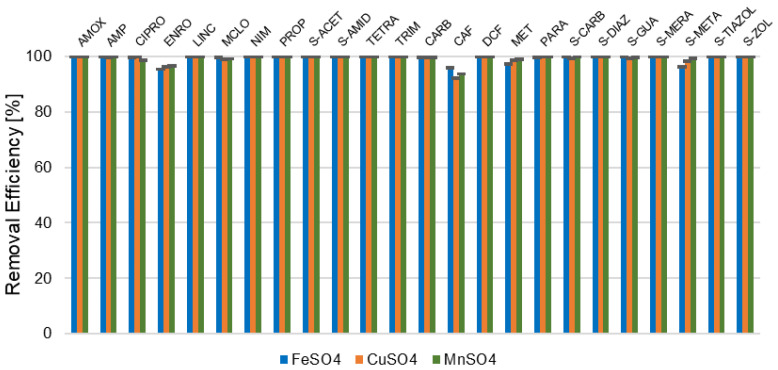
Comparative degradation efficiencies of model mixture of pharmaceutical compounds using FeSO_4_, CuSO_4_, and MnSO_4_ catalysts in Fenton oxidation (mean ± SD, *n* = 3).

The evaluation of oxidation efficiency using the Fenton process with various catalysts (FeSO_4_, CuSO_4_, MnSO_4_) for a model mixture of antibiotics and pharmaceuticals demonstrated generally high removal rates (Figure 1). Differences in degradation efficiency were observed depending on the antibiotic group and the catalyst applied.

Within the β-lactam group, both AMOX and AMP were degraded to concentrations below the method LOQ in all treatments, except for AMP with CuSO_4_, where residual levels slightly exceeded the LOQ (≈1%). For fluoroquinolones (CIPRO and ENRO), degradation efficiency varied more strongly with catalyst type, indicating higher resistance to oxidation; CuSO_4_ showed marginally higher removal compared to FeSO_4_ and MnSO_4_. Tetracycline (TETRA) concentrations also decreased below LOQ for all catalysts, reflecting their susceptibility to hydroxyl radical attack. All tested sulfonamides showed near-complete removal, with S-META showing slightly lower efficiency for FeSO_4_ (≈96%). Other pharmaceuticals (PROP, MET, CARB, CAF) exhibited moderate removal, especially with CuSO_4_ and MnSO_4_. CAF showed the lowest degradation efficiency (FeSO_4_ ≈ 96%, CuSO_4_ ≈ 92%, MnSO_4_ ≈ 94%). In contrast, DCF and PARA were reduced to below LOQ under all tested conditions. All quantitative data are reported as mean ± SD (*n* = 3), with values below LOQ presented as “<LOQ”. Appropriate statistical methods for censored data were applied where relevant.

Gas chromatography coupled with tandem mass spectrometry (GC-MS/MS) operated in SCAN mode (45–450 *m*/*z*) was used to profile organic compounds in hospital wastewater samples. This ensures non-selective detection and identification of unknown compounds. This approach may correspond to the determination of the Chemical Oxygen Demand (COD) index. Chromatogram analysis allows for obtaining detailed information on the impurity profile, which remains undetermined with traditional COD determination methods. This provides a valuable tool for identifying specific groups of compounds and assessing their potential environmental impact. This allows for the qualitative assessment of compounds that may be of key toxicological and ecotoxicological importance, even though their contribution to the total organic matter load may be small.

**Figure 2:**
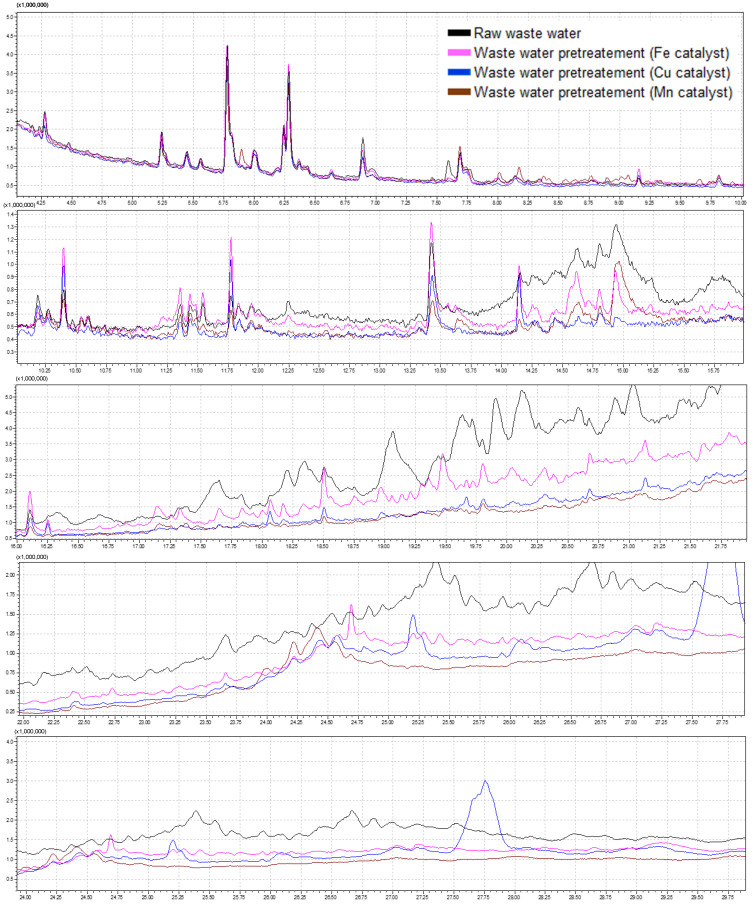
Chromatograms obtained for hospital wastewater extracts (raw and oxidated) obtained by GC-MS/MS in SCAN mode (45–450 *m*/*z*). The obtained chromatograms were divided into 5 parts according to the retention time to better visualize the differences obtained.

Chromatograms obtained for extracts from raw hospital wastewater samples revealed a high degree of matrix complexity, as evidenced by the presence of numerous peaks across the entire retention time range. Both polar and nonpolar compounds were detected, indicating the presence of a wide range of substances, from drug metabolites to disinfectants. Moreover, it was noted that retention times up to 20 min (240 °C oven temperature) showed highly similar patterns between replicates, which is presented in Figure 2. The obtained chromatograms were divided into five parts according to the retention time in order to better visualize the differences obtained.

Comparative analysis of obtained chromatograms revealed substantial differences between the chemical profiles of raw hospital wastewater extracts and those subjected to Fenton processes using various transition metal catalysts (Fe, Cu, Mn). All treated samples exhibited a general reduction in the number and intensity of chromatographic peaks, indicating partial degradation of organic contaminants. However, the extent of transformation varied significantly, depending on the metal catalyst employed. Among the tested catalysts, the iron-based Fenton process showed the highest oxidation efficiency of 70%, as evidenced by the most visible reduction in the number of detectable compounds and a significant decrease in the peak areas in the chromatogram. This suggests that iron-induced hydroxyl radical generation was more effective in breaking down organic compounds under the applied reaction conditions. In contrast, the chromatograms of samples treated with copper and manganese catalysts showed a relatively moderate reduction in signal complexity and intensity, at 36% and 39%, respectively. While some degradation of target compounds was evident, the persistence of certain major peaks indicates incomplete oxidation and lower reactivity of these catalytic systems. Overall, the GC-MS data corroborate the superior performance of the iron-catalyzed Fenton reaction in the oxidative treatment of complex hospital effluents, offering a more effective route for the removal of structurally diverse and potentially hazardous organic micropollutants.

### 2.2. Toxicity in Microtox Test

The total toxicity values, calculated using the MicrotoxOmni (version 1.18) program for all tested samples, are presented in Table 1. In general, pre-treatment samples showed lower toxicity than those after Fenton oxidation, indicating the possible formation of transformation products with higher acute toxicity. The slight negative inhibition value (−1%) observed for the untreated W3 sample suggests a minor biostimulatory effect (hormesis) or is representative of the baseline variability of the *Aliivibrio fischeri* assay, indicating no initial toxicity in this specific effluent.

### 2.3. Organic Matter Oxidation (COD Removal)

**Figure 3:**
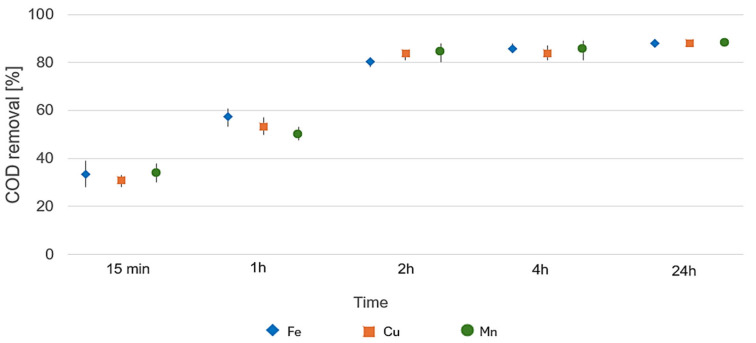
Average COD removal efficiency over time for Fenton pre-treatment of wastewater using different metal catalysts (Fe, Cu, Mn).

The overall oxidation efficiency of the Fenton process, expressed as the percentage reduction in Chemical Oxygen Demand (COD), is presented in Figure 3. The initial organic load of the raw wastewater, with average COD values around 1960 mg/L, was significantly reduced across all catalytic systems.

A rapid increase in oxidation efficiency was observed during the first 2 h of the reaction, where COD removal reached approximately 80–84%. Specifically, after 15 min, the average reduction was already above 30%, increasing to approximately 50–58% after 1 h. The highest kinetics in the initial phase (up to 2 h) were observed for the Fe-based system, though Cu and Mn catalysts showed comparable or even slightly superior performance in the later stages of the process.

Final stabilization of the oxidation process occurred between 4 and 24 h, with the average COD removal efficiency reaching approximately 88% for all tested catalysts. The final COD concentrations dropped to a range of 217–258 mg/L. These results indicate a high degree of overall oxidation of the complex wastewater matrix. The significant reduction in the total organic load, coupled with the stabilization of COD values after 4 h, confirms that the applied dosages of Fenton reagents were sufficient to transform and partially oxidize the organic pollutants present in both hospital and municipal wastewater samples.

## 3. Discussion

### 3.1. Environmental Toxicity Risk of Medical Wastewater Effluent

Hospital wastewater represents a significant source of aquatic environmental contamination, containing a wide range of chemical substances, pharmaceuticals, heavy metals, and pathogens. The concentration levels of pharmaceuticals in hospital effluents can exceed those found in municipal wastewater by two to three orders of magnitude. It is estimated that healthcare facilities contribute approximately 70–90% of cytostatic drug emissions and 30–50% of antibiotic emissions into the environment. Certain macrolide and fluoroquinolone antibiotics alone may account for up to 25% of pharmaceutical contaminants entering wastewater treatment plants. Due to their persistence, conventional wastewater treatment plants (WWTPs) often fail to effectively remove these compounds, allowing their release into surface and groundwater systems [1,16,17,18].

Pharmaceuticals used in hospitals encompass various therapeutic classes, including hormones, antibiotics, antiepileptics, beta-blockers, statins, and psychotropic drugs. Following administration, these compounds are excreted either unchanged or as metabolites, ultimately entering hospital wastewater streams [19,20]. Among them, antibiotics and cytostatics are considered the most hazardous due to their potent biological activity. Their uncontrolled release poses severe environmental threats, particularly given their potential to bioaccumulate within the food chain, leading to long-term ecological consequences [21,22,23,24].

### 3.2. Fenton Oxidation Results

The Fenton oxidation results demonstrated high overall degradation efficiency across pharmaceutical groups, with notable differences influenced by the molecular structure, catalyst redox properties, and radical interaction dynamics. Below is an enhanced discussion integrating our findings with the relevant literature.

Both AMOX and AMP exhibited complete degradation (>99%) across all catalysts, aligning with the high susceptibility of the β-lactam ring to hydroxyl radical (·OH) attack. The inherent ring strain and electron-rich carbonyl center facilitate nucleophilic assault, leading to rapid ring opening and subsequent molecular fragmentation [25]. The marginally reduced degradation of AMP with CuSO_4_ (100%) is consistent with the slower redox cycling of Cu^2+^ compared to Fe^2+^, which may limit sustained ·OH production under identical pH conditions [26]. The electron density and angle strain within the four-membered β-lactam ring likely serve as reactive “hotspots” for radical attack, emphasizing the influence of the molecular structure on degradation pathways [27].

Fluoroquinolones displayed catalyst-dependent degradation patterns, reflecting their structural resilience. CIPRO achieved full degradation with CuSO_4_ (100%) compared to 99% with MnSO_4_. Cu^2+^’s superior redox potential enhances H_2_O_2_ activation, favoring ·OH generation at a near-neutral pH [26]. The piperazine ring’s secondary amine facilitates rapid N-dealkylation and subsequent defluorination, with Cu^2+^’s coordination affinity accelerating this degradation pathway [28,29]. On the other hand, ENRO exhibited slightly lower degradation (~95–96%), influenced by its rigid bicyclic core, which necessitates sequential ·OH attacks for complete mineralization. Mn^2+^’s limited efficacy (97%) may result from Mn^3+^-induced radical recombination, reducing available ·OH species [30]. Additionally, MnO_2_ colloid formation above pH 5 may impede catalytic activity [29].

TETRA displayed near-complete degradation (>99%) across all catalysts, consistent with its polycyclic-conjugated structure, which promotes radical addition. Previous studies corroborate these findings, reporting 100% degradation under optimal Fe^2+^/H_2_O_2_ ratios. The high mineralization rate further confirms extensive oxidative cleavage of aromatic rings and N-demethylation pathways [30].

High degradation efficiencies for sulfonamides (>98%) were observed, except for S-META with FeSO_4_ (96%). This reduced performance aligns with Fe^3+^ sludge formation, which passivates active sites and limits ·OH availability. CuSO_4_ and MnSO_4_ demonstrated superior performance due to reduced sludge generation and broader pH tolerance. Degradation pathways primarily involved sulfonamide bond cleavage via ·OH attack on the S–N bond. Aromatic ring hydroxylation aligns with the literature noting high removal rates but lower COD reductions due to intermediate formation [31].

Non-antibiotic pharmaceuticals like DCF and PARA achieved complete degradation (100%), consistent with their activated aromatic structures. DCF’s dichlorophenyl group promotes electrophilic attack, while PARA undergoes rapid N-deacetylation and ring hydroxylation [26].

CAF demonstrated lower degradation rates (~92–96%), attributed to the stable xanthine ring and steric hindrance from methyl groups. FeSO_4_’s higher efficiency (96%) correlates with its faster redox cycling compared to Cu/Mn systems. PROP/MET exhibited high but incomplete degradation (~97–99%). The partial resistance is likely due to stable carboxylated intermediates and the formation of sulfoxide derivatives in MET, which resist further oxidative cleavage under standard Fenton conditions [32].

The GC-MS/MS screening provided a detailed qualitative profile of organic contaminants in hospital wastewater, complementing the bulk information obtained from COD measurements (Figure 3). While COD quantifies the total oxidizable load, chromatographic data enabled the identification of specific compound classes, including polar and nonpolar constituents, some of which may contribute minimally to COD yet hold high toxicological or ecotoxicological relevance.

The observed reduction in the number and intensity of chromatographic peaks following Fenton treatment confirms partial degradation of organic contaminants. Based on the sum of chromatographic peak areas, the Fe-catalyzed process achieved the highest removal efficiency (70%), outperforming Cu (36%) and Mn (39%) catalysts under the applied conditions. The persistence of several peaks in Cu- and Mn-treated samples suggests incomplete oxidation and lower reactivity of these systems.

These findings are consistent with previous reports on the superior hydroxyl radical generation in Fe-based Fenton reactions. Iron’s high reactivity in acidic conditions and its ability to catalyze hydroxyl radical formation effectively facilitated the breakdown of structurally diverse organic contaminants in hospital effluents. The combination of COD monitoring and GC-MS profiling proved effective for evaluating both the overall organic load and the compositional changes during treatment, highlighting the Fe-catalyzed process as the most promising approach for reducing structurally diverse micropollutants in hospital wastewater.

Although copper-based Fenton systems did not achieve the highest removal efficiency in this study, literature data indicate their significant potential, particularly under neutral pH conditions. Copper’s ability to cycle between Cu(I) and Cu(II) oxidation states facilitates effective activation of hydrogen peroxide, generating hydroxyl radicals with high selectivity and stability, which can enhance pollutant degradation efficiency [33,34]. Such systems are considered active, affordable, and stable alternatives to traditional iron catalysts, especially in configurations where acidic conditions are undesirable.

The similar number of chemical compounds detected before and after treatment indicates that the Fenton process primarily breaks down complex molecules into smaller fragments rather than achieving complete mineralization. The absence of high-molecular-weight compounds after treatment supports the effective degradation of such species, while the predominance of low-carbon-number aliphatic hydrocarbons in treated samples suggests partial oxidation and fragmentation of organic matter. These observations are consistent with reports on the limitations of Fenton processes, particularly regarding the persistence of certain low-molecular-weight degradation products [35].

Overall, these findings confirm that Fe-catalyzed Fenton reactions provide a highly effective method for pre-treating hospital wastewater, significantly reducing organic pollutant loads and transforming complex compounds into simpler forms. Recent reviews emphasize the potential of both Fe- and Cu-based Fenton systems, including photo-, electro-, microwave-, and ultrasonic-Fenton processes, for practical application in environmental remediation [33].

### 3.3. Toxicity in MICROTOX Test

Microtox^®^ analysis revealed an unexpected increase in toxicity following the application of the Fenton process, with samples shifting from low to very high toxicity levels. This phenomenon can be explained by several factors, notably the formation of toxic intermediate compounds and the presence of residual hydrogen peroxide (H_2_O_2_) or metallic ions. The Fenton reaction was terminated by neutralizing the wastewater to pH 7.0 using NaOH prior to the Microtox^®^ assay. While this adjustment effectively halts the catalytic cycle of hydroxyl radical production by precipitating a portion of the metal catalysts (particularly iron as hydroxides), it does not eliminate residual hydrogen peroxide. Since *Alivibrio fischeri* is extremely sensitive to oxidative stress, even trace amounts of unreacted H_2_O_2_ remaining in the neutralized samples act as a potent disinfectant, contributing to the observed inhibition levels near 100%. Furthermore, although neutralization at pH 7.0 promotes the precipitation of Fe^3+^ ions, other catalytic metals used in this study, such as Cu^2+^ and Mn^2+^, may remain partially soluble and bioavailable even at neutral pH. These residual ions, combined with unreacted H_2_O_2_ and incomplete oxidation by-products, form a synergistic toxic environment for the bioluminescent bacteria.

The analysis of exposure time revealed a consistent increase in toxicity between the 15 and 30 min intervals across nearly all treated samples. This trend indicates a cumulative toxic effect. The 30 min exposure allows for the sustained impact of residual oxidants and soluble metal species to further disrupt the bacterial metabolic pathways and luciferase enzyme activity, confirming that the post-treatment matrix remains biologically aggressive despite the pH neutralization.

Hydroxyl radicals, the primary oxidative species generated in the Fenton reaction, are highly reactive and capable of degrading a broad spectrum of organic pollutants [36,37,38]. However, as demonstrated in studies on sulfamethoxazole, initial toxicity often peaks before complete degradation due to the formation of aromatic amine/quinone derivatives with enhanced membrane permeability and reactivity towards bacterial luciferase enzymes [39]. In addition, Cu^2+^ and Mn^2+^ may exacerbate this by promoting alternative pathways producing metal–organic complexes or persistent quinoid species, which are more toxic to *Allivibrio fischeri* than the original antibiotics, consistent with electro-Fenton diuron oxidation where metabolite toxicity exceeded that of the parent compound [40]. Therefore, incomplete oxidation or the presence of excess hydrogen peroxide or metal ions may result in the formation and accumulation of toxic intermediate by-products. Notably, hydrogen peroxide itself is among the most potent oxidizing agents and may pose a significant ecotoxicological risk if not fully consumed during the process, raising concerns regarding post-treatment hygienization.

Additionally, residual iron ions that escaped precipitation may enhance toxicity by forming complexes with organic molecules, thereby increasing their bioavailability. The consistent rise in toxicity observed between 15 and 30 min suggests that these intermediates are not short-lived but persist in the reaction environment.

The qualitative analysis performed via GC-MS/MS and LC-MS/MS provides a chemical basis for the observed toxicity increase. While parent pharmaceuticals were significantly degraded, the chromatograms of treated samples revealed the emergence of several transformation products (TPs). For instance, the oxidation of aromatic compounds (such as diclofenac or paracetamol) often leads to the formation of p-benzoquinone derivatives, hydroxylated intermediates, and low-molecular-weight carboxylic acids. According to the literature [39,40], quinoid intermediates exhibit higher reactivity toward bacterial enzymes and higher membrane permeability compared to their parent drugs, directly correlating with the elevated inhibition observed in the Microtox^®^ assay. The fragmentation of the fluoroquinolone and sulfonamide rings, as identified in our qualitative screening, likely produced smaller, more polar metabolites, which, while contributing less to the overall COD, maintain high biological activity. This transition from complex parent molecules to more toxic intermediate metabolites explains the spike in toxicity despite the 81–89% oxidation of the total organic load.

The qualitative mass spectrometry (MS) analysis of intermediates provides further insight into the biological response observed in this study. Unlike the findings reported by Long et al. [41], where the electronic modulation of a Cu_2_WS_4_ catalyst induced a non-radical Cu(III)-mediated pathway leading to low-toxicity by-products, our classical homogeneous Fenton system likely favored a radical-dominated mechanism. The MS screening of hospital wastewater post-treatment identified fragments associated with partial oxidation, such as aromatic rings with hydroxyl or carbonyl substitutions. According to the literature and computational toxicity models used in recent studies [41], the presence of such a radical-derived intermediate, particularly those resulting from the incomplete mineralization of tetracyclines and fluoroquinolone, can exhibit higher toxicity toward aquatic organisms than the parent compounds. This difference in degradation pathways explains why our system showed a transient spike in toxicity (98% inhibition), whereas more advanced catalyst structures can achieve direct detoxification through high-valent metal species (Cu(III)).

Adamek et al. [42] further emphasized that a large molar excess of H_2_O_2_ can result in only partial conversion of the reagent, leading to its presence among the degradation products (DPs). Incomplete decomposition of H_2_O_2_ was identified as a likely contributor to the high ecotoxicity of the treated solution. From this perspective, achieving a high degree of mineralization through the Fenton or photo-Fenton process may be counterproductive due to the elevated quantity and ecotoxicity of resulting waste. In contrast, effective inactivation of antibiotics can be accomplished using only a slight excess of reagents, avoiding such drawbacks.

Similar findings on increased toxicity during the degradation of antibiotics via the Fenton process, attributing this to the accumulation of fluoroquinolone fragments, have been reported earlier [43]. This observation highlights the need for prolonged reaction times or supplementary treatment steps to ensure effective detoxification. Complete detoxification often requires longer treatment durations than those needed for the removal of the parent compounds.

In this context, Barbusiński [36] emphasized that a reduction in toxicity should be regarded as a critical measure of the success of the Fenton process. This is particularly important because the degradation of pollutants does not necessarily ensure environmental safety, and it may even lead to the formation of more toxic intermediates.

The baseline toxicity of the municipal sample (W4) can be further explained by the findings of Jagadeesan et al. [44]. Their study in Southwest England utilized high-resolution prescription data to demonstrate that pharmaceutical contamination and associated environmental risks are widespread across entire river catchments, driven by domestic consumption. This supports the observation that municipal wastewater (W4), even without direct hospital inputs, carries a significant chemical load capable of inducing biological toxicity in the Microtox^®^ assay.

## 4. Materials and Methods

### 4.1. Sampling

Wastewater samples were collected from distribution manholes at the connection points of medical facilities discharging wastewater. Sampling was conducted using a sampler into pre-prepared containers. Samples were collected in April 2024 at 30 min intervals, allowing for the determination of the average hourly flow characteristics of the wastewater. Samples were taken directly from the inflow side of the wastewater from a given medical facility, ensuring the collection of representative wastewater samples significantly affected by hospital discharge.

This study included three medical facilities: two hospitals with oncology departments (sample W1 and W2) and one hospital without such a department (W3). Control samples were taken from a municipal sewer system without medical waste pressure (W4). Each sampling point was sampled in triplicate to assess reproducibility.

### 4.2. Chemical Analysis

#### 4.2.1. Chemicals

Acetonitrile (ACN), isopropanol (IPA), and methanol (MeOH) of LC-MS grade were purchased from Supelco (USA), ammonia (p.a.) from Chempur (Piekary Śląskie, Poland), and ammonium formate with LCMS-grade formic acid (FA) of 98–100% from Sigma Aldrich (Darmstadt, Germany). Deionized water was obtained using the Hydrolab system (Straszyn, Poland). Dichloromethane (DCM, >99.9%) and methanol (99.9%) used in this study for GC-MS/MS identification procedure were of analytical grade for chromatography, and were obtained from Merck KGaA (Darmstadt, Germany). Qualitative Retention Time Index Standard n-alkane (C_7_–C_33_) mixture (certified reference material, 100–200 µg mL^−1^ in hexane) for determination of retention indices (RIs) was supplied by Restek Corporation (Bellefonte, PA, USA).

Amoxicillin (AMOX), ampicillin (AMP), ciprofloxacin (CIPRO), caffeine (CAF), caffeine (CAF-d9), carbamazepine (CARB), enrofloxacin (ENRO), lincomycin (LINC), metoclopramide (MCLO), metoclopramide-d3 (MCLO-d3), metoprolol (MET), metoprolol-d7 (MET-d7), nimesulide (NIM), paracetamol (PARA), propranolol (PROP), sulfacarbamide (S-CARB), sulfacetamide (S-ACET), sulfadiazine (S-DIAZ), sulfaguanidine (S-GUA), sulfamerazine (S-MERA), sulfamethazine-d4 (S-META-d4), sulfamethoxazole (S-ZOL), sulfamethoxazole-d4 (S-ZOL-d4), sulfanilamide-d4 (S-AMID-d4), sulfathiazole (S-THIAZOL), tetracycline (TETRA), tetracycline-d6 (TETRA-d6), and trimethoprim (TRIM) were purchased from LGC (Luckenwalde, Germany). Sulfamethazine (S-META) and sulfanilamide (S-AMID) were purchased from Sigma-Aldrich (St. Louis, MI, USA).

In the advanced oxidation process experiments for wastewater treatment, high-purity reagents were employed to ensure the precision and reproducibility of results. Hydrogen peroxide (H_2_O_2_, 35% *w*/*w*) was obtained from Supelco (Bellefonte, PA, USA), guaranteeing consistent oxidizing efficacy. Iron(II) sulfate heptahydrate (FeSO_4_ · 7H_2_O), copper(II) sulfate pentahydrate (CuSO_4_ · 5H_2_O), and manganese(II) sulfate monohydrate (MnSO_4_ · H_2_O) were also sourced from Supelco (Bellefonte, PA, USA), each of analytical grade to ensure high purity and minimal contamination. Furthermore, a sodium hydroxide solution (50% *w*/*w* in H_2_O) from Sigma-Aldrich (Germany), suitable for HPLC applications, was used to adjust pH levels throughout the experimental procedures. The use of these rigorously selected reagents was critical for maintaining the integrity of the oxidation reactions and ensuring the reliability of the experimental outcomes.

#### 4.2.2. Determination of Chemical Oxygen Demand (COD)

The overall organic load and the degree of organic matter oxidation were monitored through Chemical Oxygen Demand (COD) analysis. COD measurements were performed using Merck Spectroquant^®^ cuvette tests (Merck KGaA, Darmstadt, Germany) with a measuring range of 100–1500 mg/L or 500–10,000 mg/L. The samples were digested in a thermoreactor Merck TR 420 at 148 °C for 120 min, followed by spectrophotometric determination using the Spectroquant^®^ NOVA 6 spectrophotometer (Darmstadt, Germany). To ensure the accuracy of COD determination in the presence of residual H_2_O_2_, which is known to interfere with the dichromate method, the samples were neutralized to pH 7.0 and allowed to settle to promote the decomposition of the oxidant prior to analysis.

#### 4.2.3. The Fenton Oxidation Experiment

The initial concentration of pharmaceuticals subjected to the Fenton oxidation process was 100 ng mL^−1^ in the aqueous solution. The initial concentration of 100 ng mL^−1^ for each pharmaceutical compound was selected to ensure sufficient analytical sensitivity for tracking degradation kinetics via LC-MS/MS and GC-MS/MS systems, while also reflecting the higher concentration ranges (worst-case scenarios) occasionally reported in real hospital effluents. Following the addition of the Fenton reagent, samples were vigorously stirred for 30 min to ensure homogeneity and initiate the oxidation process. Although not within the scope of the present study, a retention time of 240 min was experimentally determined to yield the highest efficiency in COD reduction. The classical H_2_O_2_/catalyst molar ratio typically ranges from 1.2 to 2.5 mol/mol. In our study, a ratio of 1.5 was applied [31]. The influence of the H_2_O_2_/catalyst molar ratio on the process efficiency was evaluated using COD removal as the primary indicator (Figure 3). Based on these results, a ratio of 1.5 was identified as optimal for achieving maximum oxidation efficiency and was subsequently applied in all further experiments involving pharmaceutical degradation and ecotoxicological assessments. Upon completion of the oxidation phase, the mixture was neutralized using sodium hydroxide (NaOH) to achieve a final pH of 7.0–7.2. Finally, 1 mL of the oxidized solution was transferred to a vial, and an appropriate amount of isotopically labeled standards (at the same concentration as the initial pharmaceuticals) was added to compensate for the matrix effect. The oxidation experiment was also replicated using a pharmaceutical model mixture to ensure consistency and comparability of results.

The Fenton experiments were conducted at an initial pH of 3.0, which is widely recognized as the optimal value for maximizing the generation of hydroxyl radicals (∙OH) and maintaining the solubility of Fe^2+^ ions [8,30]. Operating at this pH prevents the precipitation of iron as oxyhydroxides, which would otherwise deactivate the catalyst. While recent studies [41] explore Fenton-like processes over broader pH ranges using advanced catalysts, our study focused on the comparative efficiency of different transition metals under standard, stringent conditions to ensure the maximum possible degradation of recalcitrant pharmaceuticals before assessing their ecotoxicological impact.

#### 4.2.4. LC-MS/MS Measurements

To determine the pharmaceutical removal effectiveness of the Fenton oxidation of municipal wastewater influenced by hospital effluents, the concentrations of the selected pharmaceuticals were measured before and after the oxidation experiments using a Nexera X2 liquid chromatograph coupled with an LCMS 8050 triple quadrupole spectrometer from Shimadzu Corp. (Kyoto, Japan) equipped with two pumps (LC-30AD model), an autosampler (SiL-30AC model), a thermostat (CTO-20AC), and a system controller (CBM-20A). The column used for chromatographic separation was a 150 mm × 2.1 mm × 2.6 µm Kinetex Phenyl-Hexyl (Phenomenex, Torrance, CA, USA). Measurements were performed in acidic and alkaline phase modes described in the previous study [45]. The limits of detection (LOD) and quantification (LOQ) were established in accordance with the International Council for Harmonisation (ICH) guidelines, using the standard deviation of the response and the calibration curve slope [46]. The calibration curves covered the range of 1 ng·mL^−1^ to 500 ng·mL^−1^ and consisted of nine calibration points. Details of the LOD and LOQ for measured pharmaceuticals are presented in Table 2.

#### 4.2.5. GC-MS/MS Measurements

For the chemical compound identification in wastewater samples, a gas chromatograph (GCMS-TQ8040, Shimadzu Corp., Japan) coupled with a tandem mass spectrometer (MS-TQ8040, Shimadzu Corp., Japan) and connected to an autosampler (Auto Injector AOC-20ia, Shimadzu Corp., Japan) was used in this study. The obtained wastewater samples after Fenton reaction with different catalysts, as well as raw wastewater samples, were first extracted with DCM. For 1000 mL of sample, 20 mL of solvent was used. The extraction was carried out for an hour in the orbital shaker (Stuart SI500 Incubator, 230v, Burlington, USA) at room temperature. Subsequently, the organic layers were collected and evaporated to dryness in a nitrogen stream. Next, 100 μL of DCM was added to each sample to dissolve the dry residue. The extracts were then injected to a gas chromatograph (GC-MS/MS) for qualitative analysis.

A ZebronTM capillary column (ZB-5MSi) with a length of 30 m, an internal diameter of 0.25 mm and a stationary-phase thickness of 0.25 µm (Phenomenex, Torrance, CA, USA) was used to separate the analytes. Samples were injected into the injector at a temperature of 250 °C and a volume of 2 µL. Helium (99.999995% purity) supplied by Air Products (Częstochowa, Poland) was used as the carrier gas at a constant flow rate of 1 mL min^−1^. Chromatographic separations were performed in the following temperature program: from 40 °C to 290 °C (with a ramp rate of 10 °C min^−1^) and a hold at 290 °C for 2 min. The mass spectrometer (MS) was operated in SCAN mode in the range of 45–450 *m*/*z* from the 3rd minute of the temperature program. The working conditions of the mass spectrometer were as follows: interface temperature 300 °C, ion source temperature 220 °C, ionization voltage 70 eV, emission current 150 µA. 

Chromatographic data were processed using GCMS Postrun Analysis ver. 4.45 software. Identification of compounds was performed using similarity searches in the mass spectral libraries of the National Institute of Standards and Technology (NIST 11 and NIST11s), as well as in the database of 433 pesticides (Smart Pesticides Database software (version 1.03, Shimadzu Corp., Kyoto, Japan). Moreover, the Automatic Adjustment of Retention Time (AART) program was also applied. AART is integrated within GCMS solution ver.4.45 software, increasing the accuracy of compound identification against NIST and pesticide libraries by compensating for retention time (RT) shifts resulting from column degradation, temperature changes, or complex sample matrices. For this purpose, a mixture of n-alkane standards (C_7_–C_33_) at 1 mg L^−1^ was injected and separated under the GC conditions. Then, the retention time for (C_14_–C_33_) hydrocarbons was determined and, based on this, the retention indices were automatically calculated. AART automatically recalibrate experimental RTs, aligning them with library reference values and thereby improving spectral match factors without manual intervention. The use of AART in MS full scan mode serves as an effective internal standard surrogate when authentic analyte standards are unavailable, enhancing qualitative reliability in post-run GCMS solution analysis. In addition, before the analyses, the internal tuning of MS was performed using perfluorotributylamine (PTFBA) to optimize ion source performance and mass accuracy, ensuring reliable qualitative analysis and compound identification.

### 4.3. Toxicity Tests—Microtox

Toxicity endpoints in wastewater samples were determined by assessing the inhibition of bioluminescence in *Aliivibrio fischeri* using the Microtox^®^ bioassay system after 15 and 30 min of incubation. This method is widely recognized for its sensitivity and effectiveness in evaluating the toxic impact of wastewater contaminants. The assay is based on the principle that toxic substances reduce the natural bioluminescence emitted by *A. fischeri*, with greater inhibition indicating higher toxicity levels [47]. This qualitative approach avoids the need for complex calculations while still providing reliable toxicity data. The methodology has been effectively applied in various wastewater assessments, demonstrating consistent correlation between luminescence reduction and contaminant concentration [48]. Each Microtox^®^ assay run included negative controls prepared with sterile deionized water to account for baseline luminescence levels, and positive controls using a standardized zinc solution. Quality assurance in the Microtox^®^ bioassay was ensured using a zinc sulfate reference toxicant solution, prepared according to the manufacturer’s protocol, with results normalized to the control to account for baseline variability and validate assay sensitivity. Toxicity measurements demonstrated consistent performance within the specified control limits (0.6–2.2 mg Zn^2+^ L^−1^ at 15 min), confirming compliance with standard quality control criteria for acute bacterial bioluminescence inhibition testing.

## 5. Conclusions

This study demonstrated that combining GC-MS/MS profiling with COD measurements and ecotoxicological testing provides a comprehensive assessment of wastewater treatment efficiency. The Fenton process proved highly effective for organic matter oxidation, achieving over 88% COD removal. Among the tested catalysts, the Fe-catalyzed process achieved the highest qualitative removal. A key finding is the significant post-treatment increase in toxicity (up to 98% inhibition), which remained high despite the 81–89% reduction in COD. This phenomenon, along with the toxicity increase observed between 15 and 30 min of exposure, indicates that the treated matrix remains biologically active, potentially due to persistent intermediate metabolites or residual oxidant activity. Furthermore, the similar baseline toxicity of municipal (W4) and hospital effluents suggests a widespread distribution of pharmaceutical contaminants in the sewerage system. Future work should focus on identifying specific transformation products and optimizing the quenching of reagents to ensure a genuine reduction in the environmental risk of treated wastewater.

## Figures and Tables

**Table 1:** Microtox toxicity results as the growth inhibition (in %) of *A. fisheri* for raw wastewater and pre-treatment using Fenton reaction and different metal catalysts.

Sample No.	Raw Wastewater (Before Fenton Pre-Treatment)		Wastewater Pre-Treated with a Catalyst:					
			FeSO_4_		CuSO_4_		MnSO_4_	
	(15 min)	(30 min)	(15 min)	(30 min)	(15 min)	(30 min)	(15 min)	(30 min)
W1	11%	14%	96%	97%	95%	97%	90%	89%
W2	22%	23%	91%	95%	92%	96%	91%	95%
W3	0%	−1%	91%	94%	94%	95%	90%	93%
W4	16%	18%	91%	94%	97%	98%	91%	89%

**Table 2:** Summary of method validation parameters (LOD, LOQ, and calibration curve determination coefficients (R^2^)) for target pharmaceuticals.

Pharmaceutical	Isotopically Labeled Internal Standard	Regression Equation	Linearity (R^2^)	Coefficient of Variability	LOD (ng mL^−1^)	LOQ (ng mL^−1^)
CARB	MCLO-d3	y = 0.0014x + 0.0033	1.000	0.0376	0.465	1.39
CAF	CAF-d9	y = 0.0036x + 0.0381	0.997	0.0955	2.20	6.61
MCLO	MCLO-d3	y = 0.0013x + 0.005	1.000	0.0414	0.566	1.70
MET	MET-d7	y = 0.0208x − 0.0329	0.992	0.128	7.46	22.4
PARA	PARA-M-d3	y = 0.0126x − 0.0005	1.000	0.0365	0.914	2.74
S-ACET	S-AMID-d4	y = 0.3428x − 0.1394	0.995	0.0971	0.654	1.96
S-AMID	S-AMID-d4	y = 0.0089x − 0.0181	0.996	0.301	4.78	14.3
S-CARB	S-META-d4	y = 0.0009x − 0.0021	0.999	0.0506	0.692	2.08
S-DIAZ	S-META-d4	y = 0.0011x − 0.0035	0.999	0.0627	0.784	2.35
S-GUA	S-META-d4	y = 0.0022x − 0.0019	1.000	0.0504	1.37	4.12
S-MERA	S-META-d4	y = 0.0029x − 0.0059	0.999	0.0452	0.573	1.72
S-META	S-META-d4	y = 0.0034x − 0.0035	0.998	0.0494	1.02	3.05
S-TIAZOL	S-ZOL-d4	y = 0.0029x + 0.0082	0.999	0.0452	0.461	1.38
S-ZOL	S-ZOL-d4	y = 0.0066x + 0.0136	1.000	0.0358	0.556	1.67
AMOX	MCLO-d3	y = 3 × 10^−5^x − 0.0002	0.996	0.0855	8.60	25.8
AMP	MCLO-d3	y = 3 × 10^−5^x − 0.0004	0.994	0.165	10.9	32.6
CIPRO	TETRA-d6	y = 0.0352x − 0.2461	0.998	0.253	6.80	20.4
ENRO	MCLO-d3	y = 0.0010x − 0.0028	0.998	0.0444	6.52	19.6
LINC	MCLO-d3	y = 0.0024x + 0.0068	1.000	0.0213	2.76	8.29
NIM	MCLO-d3	y = 6 × 10^−5^x + 5 × 10^−5^	1.000	0.0368	2.43	7.28
PROP	MCLO-d3	y = 0.0005x + 0.0021	0.999	0.0264	4.68	14.0
TETRA	MCLO-d3	y = 0.0002x − 0.0025	0.995	0.0326	10.2	30.7
TRIM	MCLO-d3	y = 0.0007x + 0.0025	0.999	0.0386	4.31	12.9

## Data Availability

The original contributions presented in this study are included in the article. Further inquiries can be directed to the corresponding author.
